# Effects of sex on response of the bovine preimplantation embryo to insulin-like growth factor 1, activin A, and WNT7A

**DOI:** 10.1186/s12861-018-0176-2

**Published:** 2018-07-28

**Authors:** Paula Tríbulo, Gulnur Jumatayeva, Khoboso Lehloenya, James I. Moss, Veronica M. Negrón-Pérez, Peter J. Hansen

**Affiliations:** 1 Department of Animal Sciences, D. H. Barron Reproductive and Perinatal Biology Research Program, and Genetics Institute, University of Florida, Gainesville, PO Box 110910, Gainesville, FL 32611-0910 USA; 2 Department of Agriculture, University of Zululand, KwaDlangez, Wa 3886 South Africa; 3 Present Address: Department of Innovative Technology of Reproduction and Biotechnology of Farm Animals, Kazakh Scientific Research Institute of Animal Breeding and Forage Production, Almaty, Kazakhstan 050035 / A10M6C4; 4 Present address: Department of Animal and Poultry Sciences, Virginia Tech, Blacksburg, VA 24061 USA

**Keywords:** Embryo, Sex-sorted semen, Embryokine, Insulin-like growth factor 1, Activin a, WNT7A

## Abstract

**Background:**

Alterations in maternal environment can sometimes affect embryonic development in a sexually-dimorphic manner. The objective was to determine whether preimplantation bovine embryos respond to three maternally-derived cell signaling molecules in a sex-dependent manner.

**Results:**

Actions of three embryokines known to increase competence of bovine embryos to develop to the blastocyst stage, insulin-like growth factor 1 (IGF1), activin A, and WNT member 7A (WNT7A), were evaluated for actions on embryos produced in vitro with X- or Y- sorted semen from the same bull. Each embryokine was tested in embryos produced by in vitro fertilization of groups of oocytes with either pooled sperm from two bulls or with sperm from individual bulls. Embryos were treated with IGF1, activin A, or WNT7A on day 5 of culture. All three embryokines increased the proportion of cleaved zygotes that developed to the blastocyst stage and the effect was similar for female and male embryos. As an additional test of sexual dimorphism, effects of IGF1 on blastocyst expression of a total of 127 genes were determined by RT-qPCR using the Fluidigm Delta Gene assay. Expression of 18 genes was affected by sex, expression of 4 genes was affected by IGF1 and expression of 3 genes was affected by the IGF1 by sex interaction.

**Conclusion:**

Sex did not alter how IGF1, activin A or WNT7A altered developmental competence to the blastocyst stage. Thus, sex-dependent differences in regulation of developmental competence of embryos by maternal regulatory signals is not a general phenomenon. The fact that sex altered how IGF1 regulates gene expression is indicative that there could be sexual dimorphism in embryokine regulation of some aspects of embryonic function other than developmental potential to become a blastocyst.

**Electronic supplementary material:**

The online version of this article (10.1186/s12861-018-0176-2) contains supplementary material, which is available to authorized users.

## Background

The environment established by the mother for the preimplantation embryo plays a key role in ensuring proper development. Its importance for the fate of the embryo can be observed by examination of the consequences of embryo production in vitro, i.e., in the absence of maternal signals. Such embryos differ from their in vivo counterparts in terms of gene expression [1], metabolism [2], lipid content [3, 4], ultrastructure [5], freezability [6], DNA methylation [7], competence to establish pregnancy [8] and postnatal phenotype [9–12]. One of the mechanisms by which the mother controls embryonic development involves secretion of cell signaling molecules called embryokines that modulate embryonic growth, differentiation and other aspects of embryonic function. Among the embryokines that improve development of embryos to the blastocyst stage in the cow are activin A [13], colony stimulating factor 2 (CSF2) [14, 15], insulin-like growth factor 1 (IGF1) [16], interleukin-1β [17], and WNT member 7A (WNT7A) [18].

Some alterations in the maternal environment during the periconceptional period affect developmental outcomes of female embryos differently than male embryos. Examples include consequences of in vitro embryo production [19] and maternal deficiency in dietary protein in mice [20], and feeding a diet deficient in vitamin B and methionine in sheep [21]. Sexual dimorphism in response to altered maternal function could be mediated by changes in secretion of embryokines which act on female embryos differently than male embryos. In cattle, differences in DNA methylation exist between female and male embryos as early as the 8-cell stage [22] and, by the blastocyst stage, expression of as many as one third of the expressed genes differ according to sex [23]. Experiments with CSF2 indicate that differences in cellular function between female and male embryos can lead to differential responses to cell signaling molecules. Treatment of bovine embryos with CSF2 from Day 5 to 7 of development (i.e., when the embryo transitions from the morula to blastocyst stages of development) increased the proportion of female embryos becoming blastocysts while not affecting development of male embryos [15]. Treatment of embryos with CSF2 from Day 5 to 7 also acted in a sexually-dimorphic manner to affect development subsequent to Day 15 when embryos were transferred into recipient females [24]. For female embryos, CSF2 treatment decreased trophoblast elongation and secretion of the maternal recognition of pregnancy protein, interferon-τ, but opposite effects occurred in male embryos.

It is not known whether sexual dimorphism in embryonic responses to embryokines is a widespread phenomenon or occurs for a few regulatory molecules only. To address this question, the present series of experiments were performed to determine whether three embryokines exert differential effects on female and male embryos in the cow. The three molecules studied, IGF1, activin A, and WNT7A, were chosen because genes for each of the molecules are highly expressed in the bovine endometrium during the first 7 days of the estrous cycle [25] and the proteins act on the bovine embryo to increase development to the blastocyst stage in culture [13, 16, 18].

## Results

### Development to the blastocyst stage

**Fig. 1:**
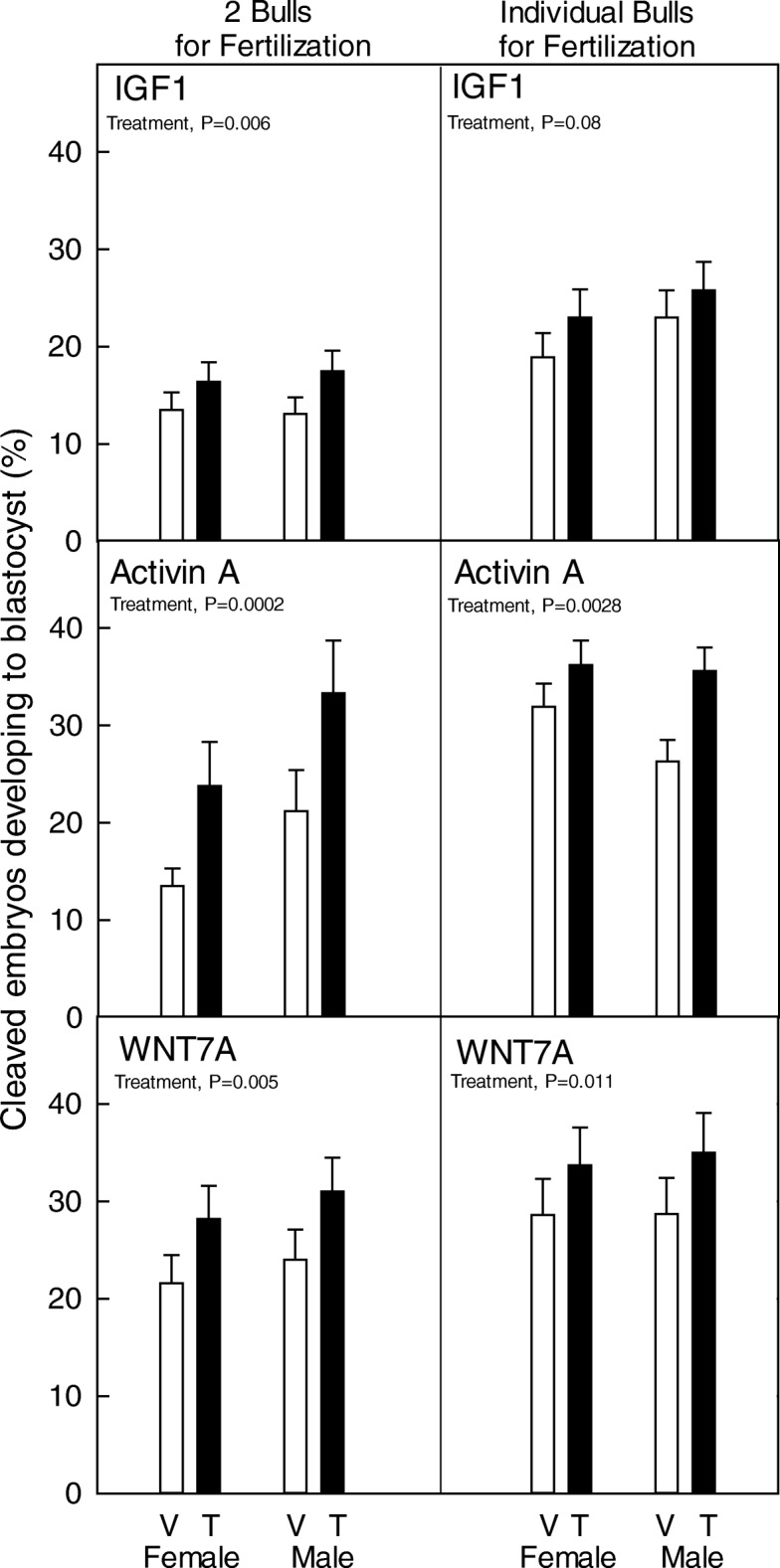
Effect of exposure of female and male embryos to embryokines (T) or vehicle (V) from Day 5 to Day 7 after insemination on the ability of cleaved embryos to develop to the blastocyst stage. Data are the least-squares means ± SEM. Significant effects are indicated in each panel

Results are shown in Fig. 1. Each of the embryokines tested increased the percent of cleaved embryos becoming a blastocyst compared to the vehicle group. This was the case for IGF1 (experiment 1, *P* = 0.006; experiment 2, *P* = 0.08), activin A (experiment 3, *P* = 0.0002; experiment 4, *P* = 0.0028) and WNT7A (experiment 5, *P* = 0.005; experiment 6; *P* = 0.011). In general, the percent of cleaved embryos becoming a blastocyst was not affected by sex. The exception was for experiment 3 in which effects of activin A were tested for embryos produced using pooled spermatozoa. In this case, a higher proportion of cleaved embryos inseminated with Y-sorted spermatozoa reached the blastocyst stage than cleaved embryos produced using X-sorted spermatozoa. There was no significant interaction between sex and treatment for any embryokine. Rather response to treatment with IGF1, activin A and WNT7A was similar for female and male embryos.

### Gene expression

**Fig. 2:**
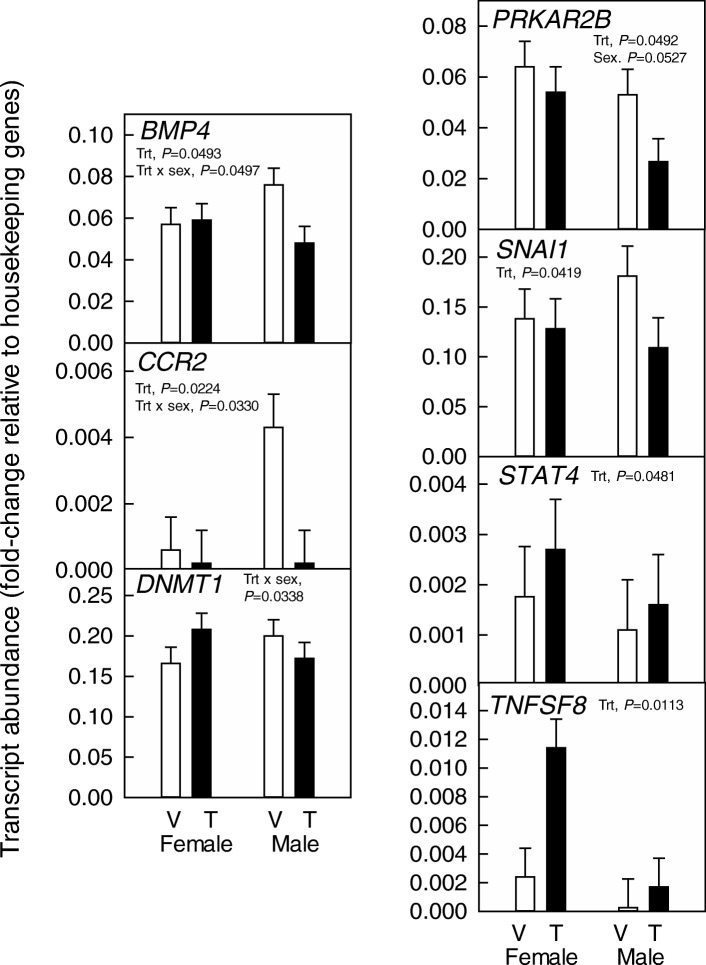
Expression of genes in blastocysts that were affected by treatment with IGF1 (T) compared to vehicle (V) from Day 5 to 7 after insemination or the interaction between IGF1 and sex. Data are least-squares means ± SEM of fold change of level of expression relative to housekeeping genes

As an additional test of whether sex affects response of the embryo to embryokines, the effect of IGF1 on gene expression of female and male blastocysts was assessed by evaluating expression using two separate 96-gene platforms. In total, expression of 127 genes was assessed (exclusive of housekeeping genes), including 53 genes for both experiments 1 and 2, 37 genes unique to experiments 1 and 37 genes unique for experiment 2. Complete results are presented in Additional file 1 and results for genes affected by IGF1 or the interaction between IGF1 and sex are presented in Fig. 2.

Expression of 18 genes was significantly affected by sex (*P* < 0.05), with 12 genes (*ACTA2*, *AKR1B1*, *AMOT*, *APOA1*, *ESRRB*, *HSD3B1*, *IFNT*, *MUC1*, *PPP2R3A*, *TGFB1*, *UBE2A*, and *XIAP*) upregulated in females and 6 genes (*CCL11*, *ELF5*, *INADL*, *MAPK13*, *ROBO*, and *SLIT2*) downregulated in females (i.e., upregulated in males). Three of the 12 genes upregulated in female embryos were on the X chromosome (*AMOT*, *UBE2A*, and *XIAP*) and none of the genes upregulated in males were located on the Y chromosome.

There were a total of 4 genes whose expression was significantly affected by the main effect of IGF1 with three genes upregulated (*SNAI1*, *STAT4*, and *TNFSF8)* and one gene downregulated (*PRKAR2B*). There were also three genes affected by the IGF1 by sex interaction (*BMP4, CCR2,* and *DNMT1*). For *BMP4* and *CCR2*, IGF1 did not change expression in females but decreased expression in males. Note that expression of *CCR2* was very low, particularly in females. For *DNMT1*, IGF1 increased expression in females but decreased expression in males.

## Discussion

None of the three embryokines tested here affected developmental potential to the blastocyst stage of the bovine embryo in a sex-specific manner. In this regard, these molecules differ from another important embryokine, CSF2. Female and male embryos respond differently to CSF2 as revealed by differences in gene expression at the morula stage [15], competence to develop to the blastocyst stage [15], and elongation of the trophoblast at day 15 of development [24]. Thus, despite differences between female and male embryos at the transcriptome level as early as the morula and blastocyst stages (present results and earlier studies [23, 26]), our data indicate that sex-dependent differences in regulation of developmental competence of embryos by maternal regulatory signals is not a universal phenomenon. Rather, it is likely that sex regulates specific cell signaling pathways so that some embryokines regulate developmental competence in a sex-specific way where others do not. The fact that sex altered how IGF1 regulates gene expression is indicative that there could be sexual dimorphism in embryokine regulation of aspects of embryonic function other than ability to develop to the blastocyst stage.

The three embryokines tested here exert their actions by downstream pathways distinct from each other. Activin A signals via SMAD proteins [27] and IGF1 activates the phosphatidylinositol 3′ kinase/AKT pathway to block apoptosis [28] and the RAS/RAF/MAP kinase pathway to promote cell proliferation, growth and differentiation [16, 29]. WNT7A can activate β-catenin-mediated WNT signaling [30] as well as the planar cell polarity pathway [31]. The cell signaling pathway for actions of CSF2 on the preimplantation embryo are not well understood because the early embryo does not express *CSFRB* [14], one of the two subunits of the CSF2 receptor. However, work with pig trophectoderm cells, in which *CSFRB* is also not expressed, indicate that CSF2 signals through phosphatidylinositol 3-kinase [32]. Since IGF1 prevents apoptosis through activation of this pathway [28] one cannot rule out that antiapoptotic actions of IGF1 are sexually-dimorphic. Indeed, there could be other aspects of development of the blastocyst not studied here, including downstream effects that occur much later in development, that could differ between female and male embryos. Embryokines like CSF2 [33, 34] and DKK1 [35] can act on the preimplantation embryo to affect fetal and postnatal phenotypes. Despite the lack of difference on development to blastocyst stage, the observation that expression of three genes was affected by the IGF1 by sex interaction is indicative that IGF1 might exert sex-dependent actions affecting other aspects of embryo function besides development to the blastocyst stage (e.g., apoptosis, allocation of cells into specific lineages, epigenetic regulation, etc.).

In general, there was no overall effect of sex on the proportion of embryos that developed to the blastocyst stage. Culture conditions, such as concentration of glucose [36] and presence of serum in the culture medium [37] can introduce a sex bias in development, which is another indication of differential susceptibility of female and male embryos to the environment. Culture conditions in the experiments here used low concentrations of glucose and the absence of serum [38].

The availability of sperm sorted based on the presence of an X or Y chromosome makes the bovine an easy-to-study species with respect to sexual dimorphism in development of the preimplantation embryo. Sex sorting of semen results in the desired sex in about 85–90% of cases [39, 40]. Sperm can be damaged by the sorting process [41, 42]. It is possible, therefore, that mixing sires for fertilization, as was done in some of the current experiments, could result in the relative number of embryos produced by particular sires being different for X-spermatozoa than Y-spermatozoa if damage did not occur equally for sperm of both type. To overcome this potential bias, all experiments were repeated using procedures where fertilization was performed with semen from a single sire only and the experiment replicated for several sires. Results were very similar whether multiple sires or single sires were used for fertilization, strengthening the idea that the embryokines tested affect male and female embryos similarly.

An important finding of these studies was the confirmation that IGF1, activin A and WNT7A enhance competence of the bovine embryo to develop to the blastocyst stage. Actions of IGF1 on the bovine embryo to increase development and block apoptosis are well described [16, 28, 43–45]. One of the few genes regulated by IGF1 in the present experiment was *TNFSF8*, which was upregulated by IGF1. Overexpression of CD30, the protein encoded by *TNFSF8*, has an anti-apoptotic effect [46]. One consequence of IGF1 treatment is increased competence of the embryo to establish pregnancy after transfer to heat stressed females [47–49]. While embryotrophic effects of IGF1 have been well characterized, this are not the case for activin A and WNT7A. Here we confirm earlier findings that both activin A [13, 50] and WNT7A [18] can increase the proportion of embryos that develop to the blastocyst stage.

## Conclusions

None of the three embryokines tested herein (IGF1, activin A and WNT7A) affected potential of the embryo to develop to the blastocyst stage in a sex-specific manner. This leads to the conclusion that sex-dependent differences in regulation of developmental competence of embryos by maternal regulatory signals is not a general phenomenon. Sex altered how IGF1 regulates expression of specific genes, however, and such a result suggests that there could be sexual dimorphism in embryokine regulation of aspects of embryonic function other than ability to develop to the blastocyst stage. Further experimentation focused on endpoints such as apoptosis, cell lineage commitment, epigenome, and development after the blastocyst stage should be conducted to clarify the situation.

## Methods

### Embryo production

Embryos were produced in vitro using oocytes harvested from ovaries recovered from a local abattoir and either X- or Y-sorted spermatozoa from Angus, Holstein or Simmental sires that were either purchased from ABS Global (De Forest, WI, USA) or Genex Cooperative (Shawano, WI, USA) or were donated by Sexing Technologies (Navasota, TX, USA). Only bulls in which both X- and Y-sorted spermatozoa were available were selected. The oocyte maturation medium was either a modified Tissue Culture Medium 199 [38] or, for experiments with WNT7A, a commercial collection medium called BO-IVM (IVF-Bioscience, Falmouth, Cornwall, UK). Media for fertilization and embryo culture were as described previously [38] except that fertilization medium contained 0.2% (*w*/*v*) amikacin sulfate (Sigma-Aldrich, St. Louis, MO, USA).

Procedures for in vitro oocyte maturation, in vitro fertilization, and embryo culture were as described previously [15, 26, 38]. Briefly, groups of 10 cumulus-oocyte complexes (COC) in 50 μL microdrops of oocyte maturation medium covered with mineral oil (Sigma-Aldrich, St. Louis, MO, USA) were matured for 22–24 h at 38.5 °C and 5% (*v*/v) CO_2_ in a humidified atmosphere. Groups of 30 matured COC were placed in 60 μl microdrops of IVF-TALP overlaid with mineral oil and mixed with 3.5 μl penicillamine-hypotaurine-epinephrine and 20 μl of sperm in IVF-TALP (final concentration ~ 2 × 10^6^/ml) that had been purified using a Puresperm 40/80 gradient (Nidacon International AB, Mölndal, Sweden). Depending on the experiments, sperm from a given replicate were either a mixture of two bulls or were from a single bull. In both types of experiments, the same bulls contributed both X- and Y-bearing spermatozoa for an individual replicate. Experiments were performed in several replicates; individual bulls or pairs of bulls differed between replicates. Fertilization was carried out for 16–18 h at 38.5 °C and a humidified atmosphere of 5% (*v*/v) CO_2_. Presumptive zygotes were washed in HEPES-TALP and then randomly placed in groups of 30 in 50 μl microdrops of SOF-BE2 covered in mineral oil. Embryos were cultured at 38.5 °C in a humidified atmosphere of 5% (v/v) O_2_, 5% (v/v) CO_2_ and the balance N_2_ or, for experiment 1, 38.5 °C and 5% (v/v) CO_2_ in humidified air. Treatments were added in a volume of 5 μl at Day 5 after insemination (i.e, 120 h after insemination). Cleavage was assessed on day 3 after insemination and blastocyst development was evaluated 7.5 d after insemination.

### Embryokine treatments

The procedure for treatment of embryos consisted of replacing 5 μl culture medium in the microdrop with 5 μl SOF-BE2 containing the embryokine at ten times the final concentration or the relevant vehicle. The final concentrations of embryokine tested were 100 ng/ml for IGF1, 1 nM for activin A and 66 ng/ml for WNT7A. Treatments concentrations were chosen because they were effective at increasing the proportion of putative zygotes becoming blastocyst [13, 16, 17]. Human activin A (amino acid sequence identity with bovine β_A_ inhibin = 95%) and recombinant human IGF1 (amino acid sequence identity with bovine IGF1 = 95%) were obtained from Sigma-Aldrich, whereas recombinant human WNT7A (amino acid sequence identity with bovine WNT7A = 99%) was purchased from eBioscience Inc. (San Diego, CA, USA). The vehicle was SOF-BE2 diluted 1:5 (*v*/v) with water for IGF1, Dulbecco’s phosphate-buffered saline (DPBS) with 0.1% (*w*/*v*) bovine serum albumin (BSA) for activin A and a mixture of 97% SOF-BE2 (v/v) and 3% (v/v) of 10 mM NaPO_4_, 500 mM NaCl and 0.5% (w/v) CHAPS diluted 1:100 (v/v) in DBPS-BSA for WNT7A.

### Experiments

A total of six experiments (IGF1, experiments 1–2; activin A, experiments 3–4; WNT7A, experiments 5–6) were conducted. In each experiment, treatment (vehicle or embryokine) was applied to 1–2 drops of embryos produced with either X- or Y-sorted spermatozoa. Each experiment was replicated on several occasions, with bulls varying between replicates. Depending on the experiment, embryos in each replicate were produced by fertilization with spermatozoa pooled from two bulls (experiments 1, 3 and 5) or with sperm from a single bull (experiments 2, 4 and 6). The total number of replicates for each experiment were 16 (10 bulls total) for experiment 1, 6 (10 bulls; experiment 2), 7 (6 bulls; experiment 3), 6 (8 bulls; experiment 4), 5 (8 bulls; experiment 5) and 7 (6 bulls total; experiment 6). In cases where the number of bulls is greater than the number of replicates, more than one bull was tested for some or all replicates.

### RNA extraction and gene expression

Blastocysts exposed to IGF1 (experiments 1 and 2) were harvested to evaluate gene expression. Immediately after collection, blastocysts were washed three times in 50 μl droplets of diethylpyrocarbonate (DEPC)-treated DPBS containing 0.1% (*w*/*v*) polyvinylpyrrolidone (PVP) and incubated with DEPC-treated DPBS-0.1% (w/v) protease from *Streptomyces griseus* for zona pellucida removal. Zona-free blastocysts were washed three times in DPBS-PVP, and transferred into RNase/ DNase-free microcentrifuge tubes, and snap frozen in liquid nitrogen. A pool of 10 blastocysts was frozen as a biological replicate for gene expression analysis. There were a total of 44 pools of blastocysts analyzed (17 for experiment 1 and 27 for experiment 2).

Each pool of embryos was subjected to RNA extraction using the Qiagen RNeasy Micro kit (Qiagen; Valencia, CA, USA); DNase treatment was included as part of the protocol. Reverse transcription was performed using the High-Capacity cDNA Reverse Transcription Kit (Applied Biosystems; Foster City, CA, USA) following manufacturer’s instructions.

The Fluidigm qPCR microfluidic device Biomark™ HD system was used to analyze gene expression using previously-described procedures [15]. The PCR primers were designed and synthesized by Fluidigm (Fluidigm Co., San Francisco, CA, USA). The set of primers for experiment 1 [51] and experiment 2 [52] were detailed elsewhere. After removing genes whose primers did not meet validation criteria, there were 37 genes analyzed for experiment 1 only, 37 genes analyzed for experiment 2 only and 55 genes that were analyzed for both experiments [two housekeeping genes, *ACTB* and *GAPDH*, and 53 other genes). Genes included those involved in cellular differentiation, apoptosis, chemokine signaling and early embryonic development.

### Statistical analyses

Effect of treatment on the proportion of cleaved embryos developing to the blastocyst stage was evaluated using Proc GLIMMIX of SAS for Windows, version 9.4 (SAS Institute Inc., Cary, NC, USA). Each embryo was considered an observation with binary response (0 = not developed to blastocyst, 1 = developed to blastocyst) and analysis was performed by logistic regression fitting binary data distribution. The statistical model included the fixed effects of treatment, sex, treatment by sex interaction and random effect of replicate (experiments 1, 3 and 5). For experiments 2, 4, and 6 bull was also included in the model as a fixed effect.

Effect of treatment on gene expression was evaluated using Proc GLM of SAS with treatment, sex and treatment by sex interaction as fixed effects, and replicate as random effect. Gene expression was calculated relative to the geometric mean of two housekeeping genes (*ACTB* and *GAPDH*). The ΔCt and 2^ΔCt^ was calculated for each gene.

For the 53 genes assessed in both experiments, data were combined and the model included treatment, sex, experiment, treatment by sex interaction, treatment by experiment interaction, sex by experiment interaction, and treatment by sex by experiment interaction as fixed effects, and replicate as random effect. When interactions were not significant, these terms were dropped from the model and the statistical analysis rerun. The response variable was ΔCt for both analyses. Results are presented as fold-change data relative to the geometric mean of the housekeeping genes (least-squares means ± standard error of the mean).

## Additional file

Additional file 1: Least-squares means of the fold-chage value for each gene examined, by treatment (control vs IGF1), sex (female vs male), and the interaction (*P*-values from dCT analyses). Tab 1: Genes measured only in Experiment 1. Tab 2: Genes measured only in Experiment 2. Tab 3: Genes measured in both Experiments 1 & 2. (XLS 95 kb)

## Abbreviations

BSA: Bovine serum albumin
COC: Cumulus-oocyte complex
DEPC: Diethylpyrocarbonate
DPBS: Dulbecco’s phosphate-buffered saline
PVP: Polyvinylpyrrolidone. Gene symbols (e.g., IGF1, WNT7A, etc.) are used as delineated at https://www.ncbi.nlm.nih.gov/gene/)
